# 

**Published:** 1901-08-15

**Authors:** 


### ANNOUNCEMENTS.

### MICHIGAN STATE DENTAL ASSOCIATION.

This Association elected the following officers June 6, 1901: President, C. H. Oakman; First Vice-President, E. A. Honey; Second Vice-President, C. C. Noble; Secretary, F. H. Essig; Treasurer, G. H. Mosher; Member Board of Censors, H. C. Raymond. The next annual meeting will be held at Grand Rapids, 1902.

